# Genetic Mechanisms of Experience-Dependent Neuronal Plasticity

**DOI:** 10.1146/annurev-genet-020325-103824

**Published:** 2025-09-17

**Authors:** Anne E. West

**Affiliations:** Department of Neurobiology, Duke University, Durham, North Carolina, USA

**Keywords:** neuron, synaptic plasticity, activity-dependent transcription, intellectual disability

## Abstract

The brain has a remarkable ability to adapt its function in response to both environmental and internal cues. The cellular composition of the brain is largely static after birth; thus, persistent experience-dependent changes in brain function depend on altered programs of gene expression that result in the plasticity of circuit connectivity and network function. High-throughput sequencing studies have comprehensively cataloged stimulus-dependent programs of gene expression in the brain. The current challenge is to integrate this information in the context of specific cells and circuits to understand the mechanisms by which transcriptional regulation coordinates adaptive plasticity of the brain and behavior. Here, I review molecular genetics studies that reveal how neuronal activity–regulated gene products orchestrate intricate cellular and intercellular adaptations in response to changes in patterns of brain activity. I also discuss examples of genetic mutations that impair experience-dependent transcriptional plasticity in the context of neurodevelopmental disorders.

## 1. INTRODUCTION

When you learn something, your brain changes. This statement is as profound as it is obvious, and for more than a century it has motivated the quest to discover the neural substrates of experience-dependent learning and memory. Over time, the methods used to probe the brain have changed, with each new approach offering answers at distinct temporal and spatial scales about the mechanisms of experience-dependent neuroplasticity.

### 1.1. Where Is Memory Stored in the Brain?

Anatomical lesion studies identified the hippocampus as essential for encoding episodic information (134). However, once learned, explicit memories are consolidated and stored diffusely in the cortex, where they are resistant to erasure following localized damage (142). The physical nature of this memory trace, also known as the engram, has come to be conceptualized as distributed ensembles of cells that are coactive during the encoding of a behavioral action and reactivated upon exposure to an internal or external cue (62). Consistent with this model, using the activity-induced expression of channelrhodopsin-2 (ChR2) to first tag and then directly reactivate ensembles of neurons is sufficient to trigger certain kinds of learned behaviors (84), whereas inhibition of the tagged ensemble can prevent the expression of some memories (32). The identification of coactive ensembles and the validation of their function as behavioral engrams have become mainstream functional anatomy approaches in systems neuroscience.

### 1.2. How Do Synapses Learn?

The concept of cellular plasticity was already appearing in the scientific literature by the late 1890s; William James used the term plasticity in a broad sense to describe how habits were learned, and then Cajal and his neuron doctrine–era contemporaries proposed that mental associations were correlates of changes in the physical connections between brain cells (94). It took another 50 years before Hebbian synaptic plasticity arose as a model for information storage among coactivated populations of interconnected neurons (51). This idea became instantiated in vivo with the electrophysiological discovery of the long-term potentiation (LTP) of excitatory synapses on neurons of the hippocampal dentate gyrus (DG) following tetanic stimulation of entorhinal input fibers (10). It is now well-established that the trafficking of AMPA-type glutamate receptors in and out of the synaptic plasma membrane is a major cellular mechanism that underlies many forms of the experience-dependent synaptic potentiation and depression that we call LTP and long-term depression (LTD) (89).

### 1.3. How Is Cellular Plasticity Integrated into Neural Circuits?

The gap that remains today is to understand how plasticity at the level of many individual cells is integrated into the changes in circuit function that underlie the learning of behaviors and the expression of memory. This is challenging to understand in the heterogeneous cellular context of the brain where different forms of plasticity occur in specific cell types over distinct timescales. Genetics has proved to be an efficient approach for defining the search space to answer this question. Gene sequencing methods have helped to define cell types, to identify the genes that show experience-dependent changes in their expression, and to discover gene mutations that are associated with impaired plasticity in intellectual disability (ID). Molecular genetics strategies in model systems provide a means to test the function of these genetic mechanisms in circuit and behavioral plasticity. Here, I review the literature investigating the functions of activity-regulated gene transcriptional programs in neuroplasticity, focusing in particular on integrative studies that seek to relate molecular/cellular with circuit/behavioral levels of understanding for experience-dependent plasticity mechanisms in the mammalian brain.

## 2. EXPERIENCE-DEPENDENT TRANSCRIPTIONAL REGULATION IN THE BRAIN

**Figure 1:**
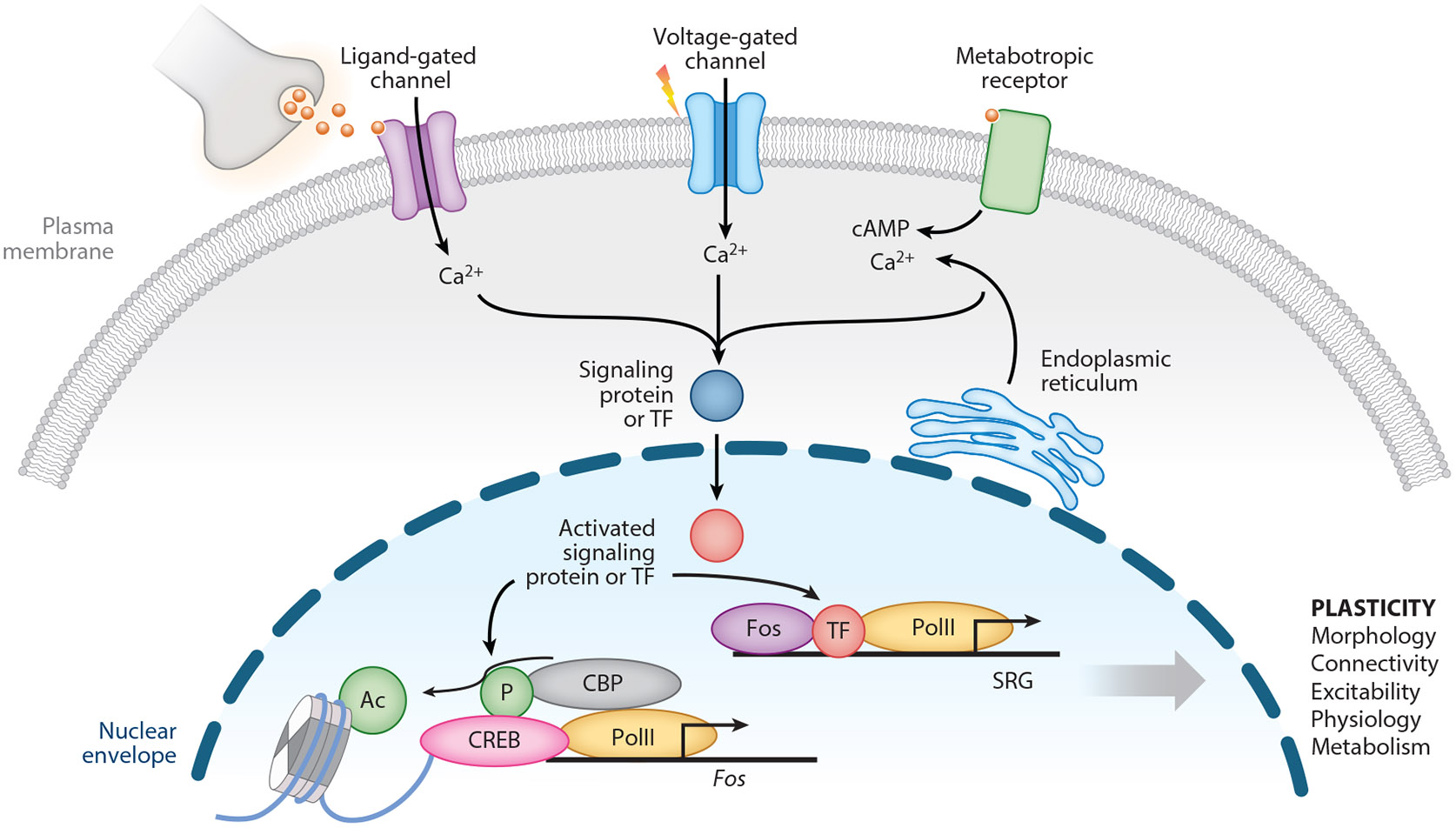
Cellular mechanisms of neuronal activity–regulated gene transcription. Neurotransmitter reception and subsequent membrane depolarization lead to the influx of calcium through ligand- and voltage-gated calcium channels at the cell membrane, as well as the release of calcium from intracellular stores by metabotropic receptors. Signaling cascades carry the signal into the nucleus, where kinases phosphorylate TFs like CREB bound to the promoters and enhancers of activity-inducible genes. Local recruitment of chromatin regulatory enzymes including CBP to these TFs promotes modification of histones and genomic DNA in chromatin. IEG transcription factors such as Fos bind to regulatory elements of SRGs that modulate synapses or cell excitability to change future responses to experience-driven neural activity (88, 176). Abbreviations: Ac, acetylation; CBP, CREB-binding protein; IEG, immediate-early gene; P, phosphorylation; PolII, RNA polymerase II; SRG, secondary response gene; TF, transcription factor.

Synaptic transmission and subsequent neuronal firing result in the activation of intracellular signaling cascades in neurons that carry signaling pathway intermediates from the synapse or plasma membrane into the nucleus (88). When these signaling pathways converge on transcription factors (TFs) and chromatin regulators, they drive significant and persistent changes in the transcription of specific genes (176). If changes in the levels of these activity-regulated gene targets are sufficient to alter aspects of neuronal physiology, morphology, or function, then this process comprises a mechanism of experience-dependent neuronal plasticity (Figure 1).

The first genes to be identified as neuronal activity–regulated are a group called immediate-early genes (IEGs), with the classic example being *Fos* (44). These genes are cellular homologs of retroviral oncogenes that got the name immediate-early because their viral counterparts are transcribed within minutes by host TFs following viral infection (74). Given their association with cellular transformation, *Fos* and other proto-oncogenes were first studied for their roles in cellular proliferation and were found to be transcriptionally induced by growth factor stimulation of fibroblasts (26, 42). It was paradigm shifting when the transcription of *Fos* was shown to be sensitive to regulation by neurotransmitters and neurotrophins, first in neuronally differentiated PC12 cells in culture (28, 43) and then in postmitotic adult neurons in vivo (102). These data first suggested that induced expression of the IEGs is a general phenomenon that serves to couple a broad range of extracellular stimuli to different kinds of biological responses, depending on the cell type (63).

IEGs are a class of primary response genes (PRGs), which means they can be rapidly transcribed in response to a stimulus without the need for prior protein synthesis. Transcriptional induction of these genes is mediated by TFs that sit prebound to regulatory elements in the promoters and enhancers of activity-responsive genes (164). In neurons, CREB, MEF2, and SRF constitute the major TF families that mediate PRG transcription (106). Posttranslational modifications such as phosphorylation of these TFs and/or their transcriptional cofactors lead to the rapid formation of regulatory complexes that both recruit RNA polymerase II (PolII) and promote active transcriptional elongation (126). Although the IEGs are in a permissive chromatin state even under basal conditions, neuronal activity (*a*) drives rapid (within minutes) promoter-enhancer looping at these genes (6), (*b*) increases histone acetylation at regulatory elements (90), and (*c*) activates the expression of enhancer RNAs, which contribute to relief of RNA PolII pausing and productive transcriptional elongation (131). Experiences that lead to learning induce specific and persistent changes in chromatin, including histone modifications (128), histone variant deposition (183), and architectural remodeling (172), suggesting that the epigenetic regulation of chromatin may contribute to the long-lasting changes in gene expression that underlie circuit plasticity and memory.

The advent of high-throughput bulk and single-cell sequencing has allowed the comprehensive identification of experience-regulated gene expression programs throughout the brain in response to a wide range of stimuli (8). The high sensitivity of sequencing compared with earlier methods of differential transcript profiling has shown that the full set of activity-regulated genes is much larger and more diverse in regulation and function than initially presumed. IEG members of the *Fos*, *Jun*, *Egr*, and *Nr4a* families are TFs that act in the nucleus. Once inducibly expressed, these TFs change cell function by binding to regulatory elements in the genome to coordinate the expression of secondary response genes (SRGs) that encode a wide range of gene products with different functions in cellular biology. Notably, in addition to the IEGs, there is a second class of neuronal activity–regulated PRGs that includes genes such as brain-derived neurotrophic factor (*Bdnf*), the signaling protein Homer1 (*Homer1a*), and others discussed below that have direct functions at synapses (77). These neuronal PRGs are also rapidly and robustly induced by sensory experience and synaptic activity, but, unlike the IEGs, they are all selectively expressed in neurons compared with nonneuronal cells. Since these proteins have known functions at synapses, changing their expression can directly alter synaptic function without the need for SRG intermediates.

One of the most important concepts to emerge from the era of transcriptional profiling is that programs of activity-dependent gene regulation are highly cell-type specific (39, 54, 93, 140, 165). This means not only that neuronal activity is restricted to regulating genes expressed in a particular cell type but also that genes expressed in more than one cell type are often only activity-regulated by any given stimulus in a subset of those cells (39). This specificity likely arises from the fundamental role of genomic regulatory elements called enhancers as modulators of statedependent programs of gene transcription (45). Enhancers are short (~500 bp) genomic regions that are binding sites for TFs. They can be a significant distance from their target genes in the linear dimension of the genome, but they are thought to form three-dimensional loops with their target gene promoters to increase the probability of RNA PolII recruitment (107). The differential activation of enhancers is a major mediator of cell type–specific differences in gene expression(52), and enhancer elements are also major targets of regulation by activity-dependent neuronal signaling pathways (68, 90).

The cell-type specificity of activity-regulated transcriptional programs is important because it means that there is not just one kind of transcription-dependent plasticity. Instead, different cells undergo distinct types of functional changes depending on the complement of genes that experience transcriptional changes. However, the functional importance of this transcriptional diversity for understanding circuit and behavioral plasticity in the brain is just beginning to be explored, as I discuss in the next section.

### 2.1. Immediate-Early Gene Transcription Factors Are Required in Specific Contexts for Experience-Dependent Plasticity

Following their initial discovery, in situ hybridization and immunostaining quickly confirmed that IEGs are induced in vivo by a broad range of plasticity-inducing stimuli. The regional patterns of IEG induction were found to vary with the stimulus and correlate with functional plasticities, providing correlational evidence for the role of the IEGs in circuit adaptations (153). Furthermore, studies demonstrated that knockout (KO) or inhibition of the rapid activity-responsive TFs CREB and SRF could impair both circuit and behavioral plasticity, suggesting that their downstream gene targets could mediate these actions (5, 36, 116, 120, 121).

An obvious next question to ask was whether IEGs are required for LTP. Among all the IEGs, the induction of *Egr1* (also known as Zif268, Krox-24, and NGFI-A) is especially well-correlated with stimuli that induce persistent LTP in the DG of the hippocampus (27). Consistent with a functional role for Egr1 in hippocampal spatial learning, constitutive *Egr1*-KO mice show slower learning and reduced memory in a water maze task (61). Basal transmission at hippocampal glutamatergic synapses is not different between *Egr1*-KO and wild-type (WT) mice, and both genotypes show normal induction of early LTP. However, *Egr1*-heterozygous and -KO mice have reduced excitatory postsynaptic current amplitudes relative to their WT siblings a day after LTP induction in vivo, suggesting that Egr1 is required for the maintenance of synaptic strengthening during late LTP in the DG. These data were consistent with prior pharmacological inhibition studies that had suggested a requirement for transcription in late LTP (104), and they helped to provide a foundation for the conceptual model that the transcriptional response to neuronal activity transduces brief experiences (learning) into long-lasting changes in circuit function (memory) (1).

However, LTP is not the only mechanism of experience-dependent plasticity, and neurons of the hippocampal DG are not the only cells that show stimulus-induced expression of IEGs. In some cases, like the example above, the functional importance of IEG induction for plasticity has been experimentally established. In other cases, such as the induction of IEGs in glial cells (33, 54), the relationship between IEG expression and cellular, circuit, or behavioral plasticity remains almost entirely unknown. In addition, just because an IEG is required for one kind of plasticity in one brain region does not mean that all correlations between IEG induction and plasticity imply causation. For example, although *Egr1* is induced in the developing visual cortex by light, *Egr1*-KO mice show normal ocular dominance shifts upon monocular deprivation during the critical period (95). In some cases, there may be overlapping or distinct functions of different members of the IEG families. Alternatively, causal relationships between IEG induction and synaptic plasticity may depend on the type of plasticity being measured. Kindling, which is a type of repeated stimulation of the hippocampus that can lead to seizure, results in the coinduction of *Egr1*, *Nr4a1*, and *Fos* in neurons of the DG along with an aberrant growth of granule cell axons in the mossy fiber pathway. Kindling-induced mossy fiber sprouting is absent in the DG of *Fos*-KO mice (162), whereas this form of activity-dependent cellular plasticity is unaffected in mice bearing KO mutations in *Egr1* or *Nr4a1* (179). In the next section, I consider how cell type–specific differences in IEG function may help to explain the variable relationship between IEG induction and resulting plasticities.

### 2.2. Immediate-Early Genes Have Cell Type–Specific Gene Targets and Functions

Because IEGs are TFs that act on cells via their induction of SRGs, understanding the relationship between IEG induction and experience-dependent plasticity requires identification of the intervening gene targets. A typical way to find the direct transcriptional targets of any TF is to conduct a genome-wide chromatin binding assay, using an antibody against the TF to immunoprecipitate or tag genomic DNA-binding sites for identification by sequencing. The heterogeneity of the brain presents a major challenge to this kind of study, because IEGs are typically induced in a fraction of each of many different types of cells within any given brain region. If a cell type of interest is identified, then that population can be purified using a cell type–specific transgene prior to conducing assays to find IEG-binding sites genome-wide (145). In addition, because IEG TFs such as Fos recruit chromatin remodelers to open chromatin at their binding sites (159), identifying genomic targets of IEGs can be possible by using methods such as assay for transposase-accessible chromatin using sequencing (ATAC-seq) to identify newly accessible regions of chromatin in purified Fos+ nuclei (38, 92, 147). Genome-wide studies of this type have shown that Fos- and Jun-containing transcriptional complexes (called AP-1 complexes) bind predominantly to accessible regions of DNA that are distal to genes and marked by acetylation of histone H3 lysine 27, all of which are characteristic of TF-binding sites called transcriptional enhancers (90, 135). These data further reveal that IEG-binding sites are highly cell-type specific and confirm a tight relationship between binding sites for IEG TFs and cell type–specific programs of stimulus-inducible gene expression (135, 159).

Even when the full set of a TF’s transcriptional targets is known, it can be challenging to identify which gene targets are biologically relevant in any given cellular context. Thus, a parallel strategy is to start by identifying plasticity changes in IEG+ neurons and then identify target genes and their expression. This has been a productive way to narrow in on biologically relevant mechanisms of IEG functions. For example, when an adeno-associated virus (AAV)-based Fos activity reporter was used to permanently label hippocampal CA1 neurons that became Fos+ during exposure to a novel environment, these neurons were found to have stronger inhibitory inputs from parvalbumin (PV)+ interneurons and weaker inhibitory inputs from cholecystokinin (CCK)+ interneurons compared with surrounding Fos– CA1 neurons (177). These inhibitory synapse changes are dependent on the expression of Fos family members because sparse AAV-Cre-mediated triple conditional KO (cKO) of all three stimulus-induced Fos/Jun family members (*Fos*, *Fosb*, and *Junb*) blocks the effect of experience on these synapses. Triple-Fos/Jun-cKO mice show impaired performance in the Morris water maze, indicating the relevance of these synaptic plasticity events for hippocampus-mediated spatial learning. To identify possible mediators of this plasticity, Yap et al. (177) performed multiple sequencing studies and identified 17 genes that show overlap between the sets of (*a*) activity-induced genes in CA1, (*b*) genes that require Fos/Jun for their expression, and (*c*) direct Fos/Jun targets as indicated by genomic binding. From this small set, RNA interference–mediated knockdown of the secreted neuropeptide precursor secretogranin 2 (*Scg2*) blocked the changes in both PV and CCK synapse strength onto Fos+ cells, suggesting that Scg2 is a key intercellular coordinator of this circuit plasticity.

These data on Fos/Jun family function in CA1 pyramidal neurons show how IEG induction in a sparse population of excitatory neurons can locally sculpt inhibitory networks via production of a secreted factor that acts in a paracrine manner to induce the plasticity of local interneurons. However, IEGs are also stimulus inducible in GABAergic interneurons, where they can directly regulate interneuron gene expression in ways that are also relevant for learning.

For example, the IEG *Nr4a1* is induced in both PV+ and somatostatin (SST)+ interneurons of the hippocampus following contextual fear conditioning (CFC) (56). However, cKO of *Nr4a1* in PV+ neurons enhances short-term memory in the CFC task, whereas cKO of *Nr4a1* in SST+ neurons impairs both short-term and long-term memory. These data show that the function of Nr4a1 in plasticity depends on the type of hippocampal interneuron in which it is induced. Recordings from CA1 pyramidal neurons in the cKO strains showed that loss of Nr4a1 weakened synaptic inhibition from PV+ cells but strengthened inhibition from SST+ neurons. These changes in synaptic strength were paralleled by changes in the number of terminals, with *Nr4a1*-cKO PV+ neurons showing fewer synaptic contacts on CA1 somata compared with WT, whereas *Nr4a1*-cKO SST+ neurons had more contacts. Transcriptional profiling showed altered expression of genes encoding axon guidance and synaptic adhesion proteins in the cKO neurons, and knockdown of the repulsive guidance cues *Sema3c* and *Epha3* in *Nr4a1*-cKO PV+ neurons rescued somatic innervation onto CA1 pyramidal neurons. Thus, in these examples where secreted proteins or cell adhesion proteins are IEG targets, transcriptional activation of IEGs in one cell can affect plasticity at the level of multicellular circuits.

### 2.3. The Activity-Induced Neuronal Transcription Factor Npas4 Differentially Regulates Inhibitory and Excitatory Synapses in Different Cell Types

The examples of *Fos* and *Nr4a1* show that IEGs can have distinct functions in synapse and circuit plasticity depending on the cell type in which they are induced. This principle is even more clear when comparing the functions of any single IEG between different cell types, as has been shown for the neuronal PAS-domain protein 4 (*Npas4*). Unlike the classic IEGs, *Npas4* is responsive to calcium signaling but not growth factors, and it is selectively expressed in neurons. However, like classic IEG TFs, *Npas4* is rapidly induced in many types of neurons following stimuli, and it is tightly associated with activity-dependent synaptic changes (150).

**Figure 2:**
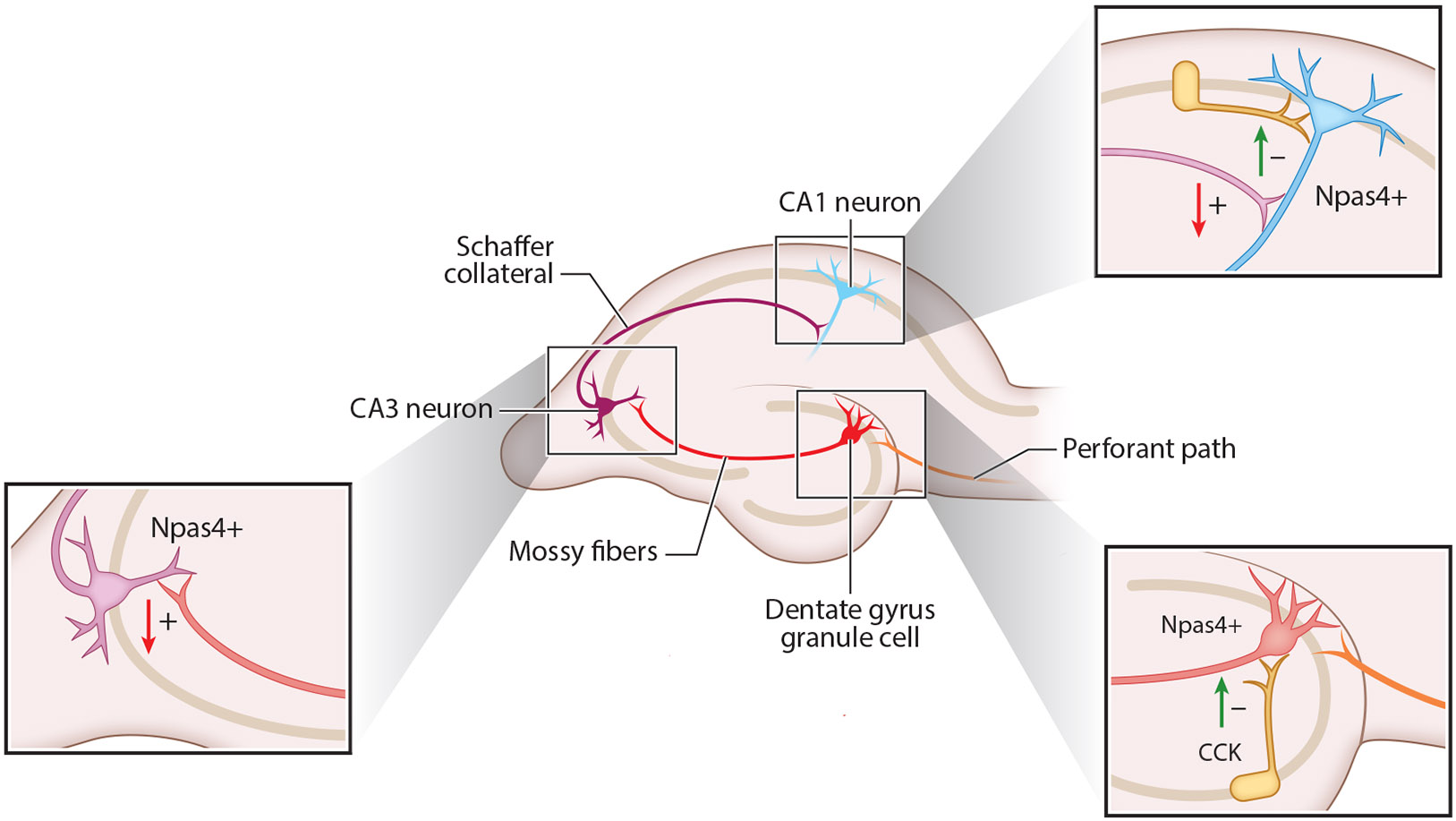
Cell type–specific synaptic functions of *Npas4* induction in the hippocampus. Npas4 is induced in DG, CA3, or CA1 neurons of the hippocampus in response to different stimuli. In each case, induction of Npas4 (Npas4+) acts to decrease excitatory drive to neurons but via distinct effects on excitatory and inhibitory synapses. In DG, Npas4 drives increases in inhibition from CCK+ interneurons. In CA3, Npas4 inhibits the strength, size, and number of excitatory inputs from the mossy fibers. In CA1, Npas4 both increases inhibitory synapse number and decreases excitatory drive (81, 149, 163). Green arrows indicate an increase in number or strength, while red arrows indicate a decrease in number or strength. Plus symbols indicate excitatory synapses, and negative symbols indicate inhibitory synapses. Abbreviations: CCK, cholecystokinin; DG, dentate gyrus; Npas4, neuronal PAS-domain protein 4.

Npas4 was first characterized for its ability to promote inhibitory synaptogenesis onto excitatory hippocampal neurons during development (81). Lin et al. (81) found that Npas4 is not required for the initiation of excitatory synaptogenesis, but experimentally increasing the levels of Npas4 after synapse formation decreases excitatory synaptic input to the same cell. This evidence that increasing Npas4 in CA1 neurons enhances inhibitory synapses relative to excitatory synapses suggests that Npas4 serves as a cell-autonomous negative feedback mechanism in active neurons to reset the excitatory/inhibitory (E/I) ratio (Figure 2). The biological importance of this homeostatic plasticity function in excitatory neurons has been demonstrated in the visual cortex, where the daily light-driven induction of Npas4 is required to maintain the stability of cortical representations (72).

Even within the hippocampus, the specific actions of Npas4 on synapses vary among different excitatory neurons. During CFC, *Npas4* is induced in select excitatory neurons of the DG and CA3, and its induction in these neurons is required for encoding of contextual memory (119, 149). In the DG, Npas4 acts to increase inhibitory inputs onto excitatory neurons specifically from CCK+ interneurons (149). However, in CA3, Npas4 functions primarily to restrain excitatory input onto these neurons rather than increasing inhibition (163). Specifically, cKO of Npas4 in CA3 neurons results in a selective increase in the number, size, and strength of mossy fiber CA3 excitatory synapses. Weng et al. (163) identified the Polo-like kinase *Plk2* as an Npas4 target gene in CA3 neurons that functions postsynaptically to restrain synapse growth. They proposed that the memory deficits in *Npas4* CA3 cKO mice arise because the aberrant increase in mossy fiber to CA3 connectivity occludes the formation of the sparse ensemble in CA3 needed to encode the spatial memory (163).

These examples show that Npas4 can shift the E/I ratio in excitatory neurons toward inhibition by increasing inhibitory synapses, decreasing excitatory synapses, or both. However, Npas4 is also induced by neuronal activity in inhibitory neurons, raising the question of whether it has cell-type differences in its function there (140, 173).

In developing SST+ GABAergic interneurons, knocking out Npas4 had no effect on inhibitory synapses but decreased excitatory synapses (140). Inversely, overexpressing Npas4 in SST+ neurons increased excitatory synapse number without affecting inhibitory synapses. This indicates that in these neurons at the cellular level, Npas4 promotes excitatory synaptogenesis onto SST+ interneurons. This is the opposite of the developmental function of Npas4 in excitatory neurons where Npas4 promotes inhibitory synaptogenesis. However, at the level of local neural circuits, both of these cellular effects are homeostatic.

Yang et al. (173) found that, in the adult brain, cKO of Npas4 in SST+ interneurons of the motor cortex disrupted motor learning, suggesting that Npas4 plays crucial circuit plasticity roles following its induction in inhibitory as well as excitatory neurons. The expression of Npas4 in SST+ interneurons was required for motor learning–induced plasticity of spines on excitatory projection cells, confirming a multicellular, circuit-level consequence of Npas4 regulation in a single cell type. However, unlike the proexcitatory role of Npas4 in SST+ neurons during development, Yang et al. (173) found that Npas4+/SST+ neurons showed reduced activity measured by in vivo calcium imaging across the period of motor learning. This result could represent distinct functions of Npas4 in different types of SST+ neurons or in different stages of brain development. For example, in contrast to the developmental study from Spiegel et al. (140), Kim et al. (67) reported finding reduced inhibitory synapses, and no effect on excitatory synapses, which they recorded from SST+ neurons of the CA1 region of the hippocampus in Npas4-floxed/*Sst*-Cre mice. Alternatively, any direct actions of Npas4 on synapse numbers may trigger secondary adaptations that result in changes in cellular excitability. For example, using a motor learning task, Yang et al. (173) reported changes in motor cortex network activity and behavioral consolidation over a period of 12 days. Increasingly sophisticated molecular genetic methods that control the timing of gene manipulations will help to reveal and dissect the temporal ordering of interconnected network plasticities in the future (see the sidebar titled The Promise and Perils of Using Immediate-Early Genes to Define Engrams).

### 2.4. Integrative Cellular and Circuit Functions of Activity-Induced, Neuronal-Selective Genes

Fos, Nr4a1, and Npas4 are TFs, which explains how they can regulate different programs of gene expression in different cell types, resulting in distinct forms of circuit plasticity. However, the direct synaptic actions of the neuronal-selective PRGs (e.g., *Arc*, *Bdnf*, *Cpg15*, *Homer1a*, *Nptx2*) initially suggested that these gene products might have simple, direct impacts on plasticity. The reality has turned out to be more interesting, as studies reveal that these gene products can integrate multiple kinds of plasticity via both intracellular and intercellular actions in the brain. Below, I discuss examples from the literature on Arc and Nptx2 to show how the changes in expression of these single proteins can initiate intricate circuit plasticities.

#### 2.4.1. Arc orchestrates plasticity across synapses.

Arc, the activity-regulated cytoskeletal-associated protein, is encoded by a neuronal-selective gene whose induction in the hippocampus is tightly linked to plasticity-inducing stimuli (85). Using in vivo high-frequency activation of perforant path inputs to the DG, which is the classic way to induce LTP at these synapses, Steward et al. (143) found not only that *Arc* mRNA is robustly upregulated but also that both newly synthesized *Arc* messenger RNA (mRNA) and Arc protein localize to a region of the postsynaptic granule cell dendrites that corresponds to the activated inputs. Knocking out *Arc*, either constitutively or conditionally in the hippocampus, impairs long-term memory in spatial learning tasks, suggesting that Arc is a synaptic mediator of the circuit adaptations that underlie consolidation of these memories (118, 137).

Despite these clear links between Arc and memory in canonically studied hippocampal circuits and memory paradigms, at the cellular level, multiple studies show that the induction of Arc drives endocytosis of AMPA-type glutamate receptors, which promotes LTD rather than LTP (73). Experimentally changing the levels of Arc inversely regulates surface AMPA-type glutamate receptor expression, suggesting that Arc induction has the direct homeostatic function of weakening synapses (122, 138). Mechanistically, this is mediated by an association of Arc with endophilin and dynamin, two regulators of receptor endocytosis (24). In addition, in the highly organized, protein-dense environment of dendritic spines, Arc promotes endocytosis by competing for binding to AMPAR-stabilizing proteins such as stargazin (Cacng2, TARPγ2), thus dispersing AMPARs from phase-separated clusters (22). During development, the excitatory synapse–weakening functions of Arc are required for activity-dependent elimination of synapses at the parallel fiber-Purkinje synapse in cerebellum (98) and for mGluR5/MEF2-dependent synapse elimination in hippocampal area CA1 (166).

Interestingly, at the level of a single dendrite, Arc-mediated endocytosis of receptors at selected synapses organizes patterns of synapses with different kinds of synaptic plasticity. For example, ongoing Arc-dependent AMPAR endocytosis is required to support synapse-specific homeostatic upscaling at sparse, less active synapses, presumably through redistribution of the endocytosed receptors (7). With respect to Hebbian plasticity, El-Boustani et al. (35) showed that Arc is required for heterosynaptic plasticity of spines in a model of spike timing–induced plasticity in the visual cortex. They used in vivo imaging to identify dendritic spines that showed structural plasticity in LTP/LTD (sLTP/sLTD) when ChR2-mediated spike timing was used to pair neuronal spiking to a receptive field visual stimulus. This pairing induced rapid, sustained sLTP of a small number of spines followed by a slower sLTD of other spines. The depressed spines were significantly enriched near the potentiated spines; thus, these data reveal both a temporal and spatial relationship between the two Hebbian plasticity processes. Knocking down *Arc* disrupted the receptive field shift induced by pairing, showing that Arc is required for this form of circuit plasticity. However, Arc loss had no effect on the ability of the stimulus to induce sLTP. Instead, Arc knockdown neurons had fewer overall spines with sLTD, and, unlike in WT neurons, the sLTD spines were not enriched in proximity to the sLTP spines. These data suggest that Arc induced at the activated spines acts in a heterosynaptic manner to remove AMPARs from nearby inactive spines. Indeed, it has been shown that when Arc translation is locally induced in dendrites following synaptic activity, the newly synthesized Arc protein preferentially accumulates at inactive synapses through a high-affinity interaction with calmodulin-free CaMKIIβ (111).

This role of Arc in spatially organizing synapse plasticity is predicted to be especially important in contexts where there is heterogeneity of different types of inputs along a single dendrite that are not likely to be coactive (35, 111). This could have computational significance for the cell by creating a local enhancement of the signal-to-noise ratio for information coming from the potentiated synapses. This would be similar at the dendrite level of integration to how stimulus-dependent changes in the number of silent synapses are proposed to contribute to cellular ensemble formation in regional circuits (169). How and whether the synaptic functions of Arc contribute to forms of transcription-dependent plasticity that arise from the regulation of silent synapses, such as cocaine-induced plasticity in the nucleus accumbens (13), remain a topic for further exploration.

I have focused on the AMPAR-regulatory functions of Arc because this is its most well-characterized cellular action and because AMPAR trafficking serves as a major mechanism for the plasticity of excitatory synapse strength (89). However, Arc has been implicated in additional processes in neurons, and it is likely that some of these also contribute to activity-dependent plasticity. For example, one group has shown a role for Arc in linking NMDA-type glutamate receptors to downstream regulation of the kinase Akt, with the ultimate effect of protecting potentiated synapses from subsequent depotentiation (174). Beyond the synapse, it is well-established that activity-induced Arc protein localizes to the nucleus in addition to its distribution in dendrites (11, 70); however, no one has yet selectively disrupted the nuclear trafficking of Arc to determine its specific functions in this cellular compartment. Arc induction in the context of plasticity-inducing stimuli is also likely to lead to secondary consequences on physiological features. For example, Arc+ neurons become hyperexcitable in the nucleus accumbens following repeated cocaine administration (151), and they exhibit increased correlated activity with other Arc+ neurons in culture (60). The mechanisms of these excitability changes, and how they relate to Arc induction, remain unknown.

Finally, because Arc is an intracellular protein that is induced predominantly in projection neurons, it is not surprising that the majority of studies have focused on its cell-autonomous functions. However, recent studies show that Arc can also form viral-like particles capable of delivering *Arc* mRNA and Arc protein from an activated neuron to neighboring cells (3, 113). These data raise the possibility that Arc could have intercellular actions (148). Phylogenetic sequence analysis suggests that Arc derived from a retrotransposon Gag protein in the *Ty3*/*gypsy* family and that Arc retains the ability to form viral-like capsids (48). The relative importance for plasticity of Arc’s intracellular postsynaptic functions versus its role after intercellular transfer remains poorly understood. Some clues have emerged from genetic studies of Arc in *Drosophila*, where secreted Arc made in motor neurons impacts synapse plasticity across the neuromuscular junction (3). However, *Drosophila* and mammalian *Arc* are derived from independent retrotransposons, and as a result have important differences in their functions. Future studies that selectively disrupt the ability of Arc to traffic between cells without impairing its function within cells have the potential to identify new roles for Arc in circuit plasticity.

#### 2.4.2. Nptx2 couples neuronal activity with excitatory synapses on GABAergic interneurons.

Another gene product that is robustly induced by synaptic activity in glutamatergic neurons yet has circuit-level actions is the secreted neuronal activity–regulated pentraxin (Narp) encoded by the *Nptx2* gene. There are three pentraxins expressed in the brain: *Nptx1*, a secreted pentraxin that is expressed selectively in the brain (133); *Nptx2*, which is more widely expressed but robustly induced by synaptic activity in cortical and hippocampal glutamatergic neurons (154); and *Nptxr*, the only membrane-bound neuronal pentraxin, which is proposed to anchor the pentraxins in a complex on the cell surface (76). Nptx2 binds directly to the extracellular N-terminal domain of AMPAR subunits, and all three of the neuronal pentraxins have been experimentally implicated in the clustering and/or stabilization of AMPARs at glutamatergic synapses (18, 23, 76, 109, 171).

Within cortical networks, Nptx2 plays an especially important role for the formation and stabilization of glutamatergic synapses onto GABAergic interneurons (15, 115). The subsequent modulation of inhibitory network activity is critical for cortical plasticity. In vivo, constitutive deletion of *Nptx2* decreases excitatory drive to PV+ interneurons in the developing visual cortex, resulting in both cortical hyperexcitability and an absence of experience-dependent ocular dominance plasticity (47). Ocular dominance plasticity is rescued by increasing the activity of PV+ interneurons in the *Nptx2*-KO mice (47), consistent with the essential role of local interneuron activity in regulating critical period plasticity of the visual circuit (53).

Rapid (within one day) visual deprivation-induced plasticity of ocular dominance after the critical period is also gated by Nptx2-mediated cortical inhibition, showing that this is a function of the activity-dependent regulation of Nptx2, not a developmental effect (136). The requirement for Nptx2 in AMPAR stabilization on PV+ neurons and cortical plasticity is reminiscent of the role played by perineuronal net (PNN) proteins, which are also activity-regulated both during development and in the adult brain to effect network plasticity (37). The chondroitin sulfate proteoglycans that comprise PNNs cluster around the soma and proximal dendrites of PV+ interneurons. Similar to Nptx2, the activity-dependent emergence of PNNs in the cortex during development is required for the development of excitatory synaptic inputs onto PV+ neurons and for the closure of developmental critical periods of plasticity (124). Notably, Nptx2 interacts directly with PNN components and may be part of a larger, multiprotein extracellular matrix regulatory mechanism of neural circuit development (158).

**Figure 3:**
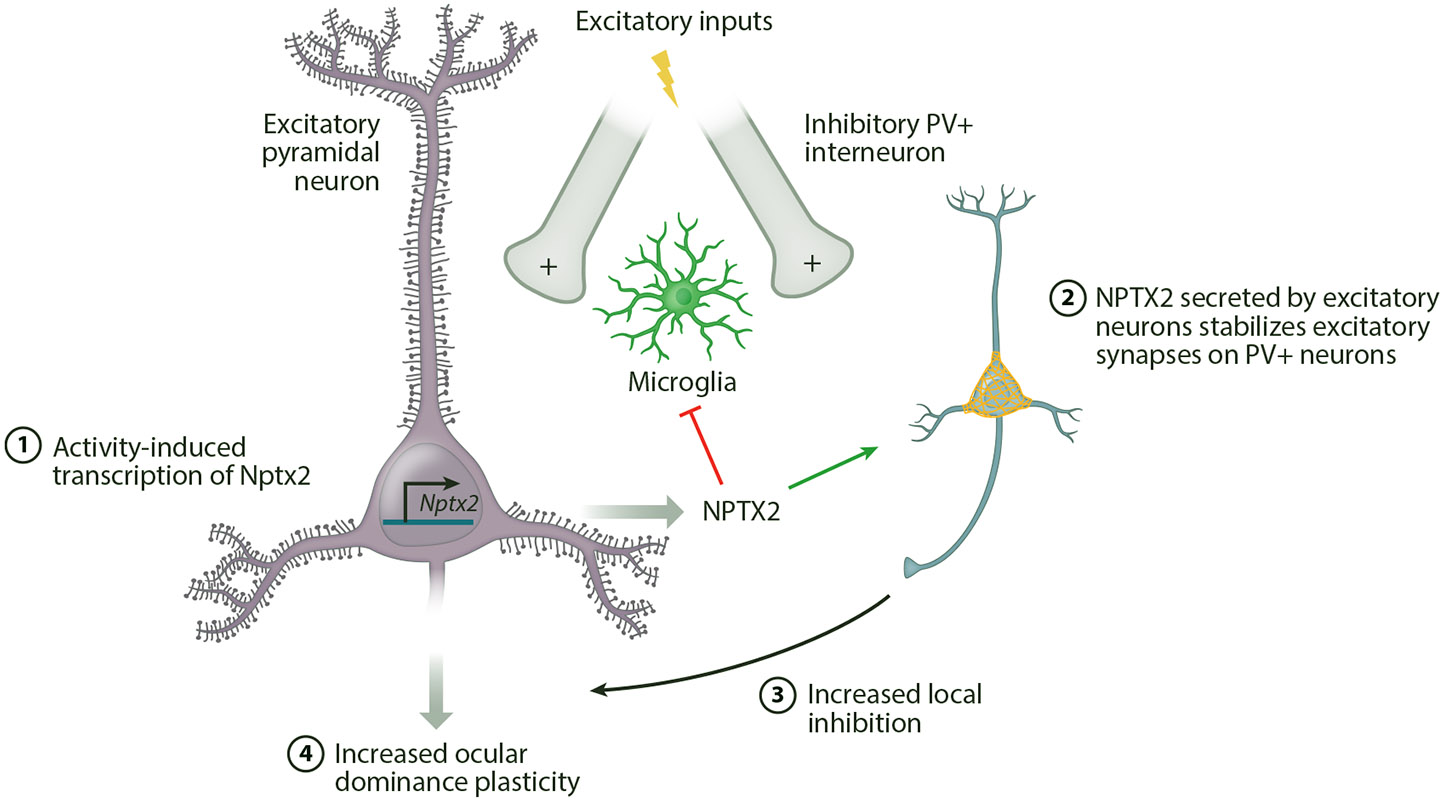
Nptx2 has intricate effects on network connectivity. The activity of excitatory inputs to the cortex leads to the production and release of Nptx2, which accumulates at excitatory synapses onto inhibitory parvalbumin (PV)+ interneurons. Nptx2 stabilizes AMPARs at these synapses. The increased excitatory drive to PV+ cells inhibits cortical excitatory neurons, permitting ocular dominance plasticity (136, 180). Nptx2 may also actively prevent microglial engulfment of excitatory synapses by blocking C1q.

These data suggest a structural role for Nptx2 in excitatory synapse stabilization. However, Nptx2 may also play a more active role in preventing excitatory synapses from being eliminated (Figure 3). Outside of the neural plasticity context, pentraxins are best known for their roles as pattern recognition receptors that regulate innate immunity. Consistent with these proteins playing a similar innate immune regulatory role in the central nervous system (CNS), neuronal pentraxins bind to the complement factor C1q in the brain (71, 144). During development, C1q tags synapses for elimination by activating C3 to stimulate microglial engulfment in a process that is required for developmental plasticity of cortical wiring (130). C1q also contributes to synapse loss in neurodegenerative disorders (167). The interaction of C1q with Nptx2 constrains the ability of C1q to activate the complement cascade, ameliorating microglia-mediated excitatory synapse loss in a neurodegenerative disease mouse model (180). This means that activity-induced expression of Nptx2 has the potential to serve as a negative regulator of complement-mediated synapse elimination both in development and in aging. Interestingly, NPTX2 is detectable in cerebral spinal fluid, and reduced levels of NPTX2 have been reported to serve as a sensitive biomarker for impaired cognitive function in a variety of neurodegenerative pathologies, including in Alzheimer’s disease, frontotemporal dementia, and Parkinson’s disease (105, 157, 170). In addition to providing a potential target to report the synaptic actions of disease-modifying therapeutics, the activitydependence of Nptx2 nominates it as an intriguing candidate to mediate the neuroprotective effects of cognitive activity in the aging brain.

## 3. CONTRIBUTIONS OF ACTIVITY-REGULATED GENES TO INTELLECTUAL DISABILITIES

ID emerges during childhood and is defined by differences in two core domains of function: (*a*) intellectual function, which includes the ability to learn, reason, and solve problems, and (*b*) adaptive behavior, which describes the ability of the individual to carry out everyday life and social skills. ID is often found together with other kinds of neurodevelopmental disorders, including autism spectrum disorder (ASD), epilepsy, and mental health conditions (75). In parallel with the substantial clinical overlap between these diagnoses, there is also genetic overlap, such that mutations in a single chromosomal region or gene can be associated with more than one clinical presentation (160).

Most cases of profound ID arise from genetic causes, and the elucidation of the genetic architecture of ID has rapidly accelerated in conjunction with the development of methods with increasingly fine chromosomal resolution (91). Large chromosomal disruptions visible in karyotyping gave the first clues about the genes that underlie ID, with trisomy of chromosome 21, which causes Down syndrome, as the most common genetic form of ID (125). The introduction of microarray-based methods for comparative genomic hybridization (aCGH) accelerated the discovery of microdeletions and duplications in patients with ID (80). More recently, the widespread application of exome sequencing has radically advanced the identification of rare, de novo variations in single genes that have a strong effect on cognitive phenotypes. As of January 2025, the curated database SysNDD (69; https://sysndd.dbmr.unibe.ch/) reports 1,736 genes with a definitive association to neurodevelopmental disorders, which include developmental delay, ASD, and ID. Mechanistic studies of ID-associated genes in model systems offer the opportunity to discover the neural basis of cognition, including the role of these gene products in experience-dependent transcriptional plasticity.

### 3.1. Disruption of Activity-Dependent Gene Regulation in Developmental Brain Disorders

The power of human genetics data is that they can reveal which molecular mechanisms are directly related to an observed phenotype. Here, I review studies that have used the association of gene mutations with neurodevelopmental disorders (NDDs) to ask about the role of activity-dependent gene regulation in the context of ID. These studies (*a*) show the cognitive impacts of mutations in core components of activity-dependent transcriptional pathways, (*b*) reveal new plasticity functions for transcriptional and chromatin regulators, and (*c*) give insights into the noncoding genome. Taken together, these data offer strong evidence for the critical molecular mechanisms that link neural activity with transcription-dependent plasticity in the human CNS.

#### 3.1.1. Neurodevelopmental disorders caused by mutations in core activity-regulated signaling components.

Activity-dependent changes in gene transcription begin at the synapse, where neurotransmitter reception triggers calcium influx through NMDA-type glutamate receptors and L-type voltage-gated calcium channels (L-VGCCs) (176). Mutations in the L-VGCC α-subunit *CACNA1C* cause Timothy syndrome (TS), a rare genetic disorder first studied as a cause of cardiac long QT syndrome, which also has neurological features including developmental delay, autism, seizures, and ID (141). TS point mutations in the intracellular loops of the Ca_v_1.2 α-subunit have complex effects on the dynamics of calcium influx through these channels as well as impacting structural, channel-independent signaling mediated by Ca_v_1.2 (78). Induced pluripotent stem cell (iPSC)-derived neurons from patients with TS mutations show changes in activity-regulated coexpression networks that are mediated by activation of the NFAT, MEF2, CREB, and FOXO TFs, indicating impairments of activity-regulated transcription in the presence of the mutant *CACNA1C* channels (152).

Second messenger pathways transduce calcium signals into the activation of kinases that travel to the nucleus and phosphorylate or otherwise activate stimulus-regulated TFs, including members of the CREB, MEF2, and SRF families. Among these TFs, haploinsufficiency of the MEF2 family member *MEF2C* has been identified as a cause of syndromic ID characterized by severe ID, seizures, speech deficits, neurobehavioral issues, and epilepsy, as well as cardiac manifestations (108). In mice, conditionally knocking out *Mef2c* either brain-wide or in excitatory neurons changes excitatory synapse formation and disrupts E/I ratios (4, 50). Conditional deletion of *Mef2c* in neurons is sufficient to induce deficits in learning and memory (4), disrupt cortical development (79), and alter behaviors relevant to ASD and ID (50). Some of these synaptic and behavioral phenotypes are also present in germline *Mef2c* heterozygous mice, which supports the use of this mouse model to understand the cognitive effects of *Mef2c* haploinsufficiency in humans (49). Recently, geneticists have found disease-associated mutations in the genomic regulatory regions near *MEF2C* and other NDD genes, raising the possibility that noncoding as well as coding mutations could result in ID via transcriptional dysregulation of *Mef2c* (101, 168). Indeed, human iPSC-derived models of both regulatory and coding mutations in *MEF2C* show convergent effects on genes involved in neurodevelopment and display disrupted excitatory synapse connectivity (101). These data show the importance of regulating the levels of *MEF2C* expression for brain development and cognitive function.

Phosphorylation of activity-regulated TFs drives transcription via the recruitment of the transcriptional coactivators CREB-binding protein (CBP) and p300 (25). CBP and p300 are both enzymes that acetylate histones to promote chromatin accessibility and transcriptional activation (110). CBP is recruited to activity-induced promoters and enhancers genome-wide following neuronal membrane depolarization (68), and the induction of histone acetylation at these regulatory elements has been both correlated with (90) and shown to directly modulate (19) the transcriptional induction of coupled activity-regulated genes. CBP is also phosphorylated in an activity-dependent manner at a site that facilitates its transcriptional coactivator functions (55, 59). Heterozygous loss-of-function mutations in the gene encoding CBP (*CREBBP*) cause Rubinstein–Taybi syndrome, which is characterized by distinct facial features, broad toes and thumbs, and ID (117). Some forms of this syndrome are also associated with mutations in *EP300*, the paralog of *CREBBP* that encodes p300 (123). The functions of CBP and p300 in cell survival and fate maintenance are partially overlapping in neurons (82). By contrast, only conditional KO of *Crebbp* but not *Ep300* in excitatory neurons of the adult mouse forebrain impairs both activity-dependent transcription in an enriched environment and learning in the Morris water maze, thus suggesting a privileged role for CBP in the plasticity functions of these related coactivators (83).

#### 3.1.2. Activity-dependent regulation and functions of neurodevelopmental disorder–associated chromatin regulators.

The word chromatin describes the complex of genomic DNA and its associated histone proteins that gives structural and functional context to genes in the genome. Epigenetic modifications that are laid down on chromatin, such as DNA methylation and histone modifications, alter protein complex recruitment to gene regulatory sites, changing the dynamics of gene transcription (107). Although chromatin regulatory proteins play a role in all transcription in all cells, mutations in some chromatin factors are selectively associated with NDDs (129), suggesting their potential roles in neuroplasticity.

The BAF complex contains ATP-dependent chromatin remodelers that reposition nucleosomes, thus changing chromatin accessibility to facilitate TF binding. BAF factors seem to be especially important in CNS functions because this family harbors a disproportionate number of the mutations in nuclear protein complexes that are associated with NDDs (156). Fos recruits the BAF complex to mediate the selection of cell type–specific enhancer repertoires during neuronal differentiation (159), and BAF also contributes to experience-dependent plasticity in the adult brain. For example, heterozygous KO of *Act6b*, the gene encoding BAF53b, impairs synapse plasticity and learning and memory, but these deficits can be rescued by reexpression in the adult hippocampus, showing a role for BAF53b in mature neurons after the period of development (161). The BAF component BRG1 is phosphorylated by CaMKII at a site that is required for the induction of activity-dependent genes, suggesting a direct link between BRG1 regulation and transcriptional plasticity (65).

DNA methylation is also important for cognitive function. Loss-of-function mutations in the X chromosome gene encoding the transcriptional repressor and methyl-DNA binding protein reader *MECP2* cause Rett syndrome (RTT), the most common genetic cause of ID in females (2). The postnatal timing of neurological decline in RTT correlates with the period of activity-dependent refinement of synapses in the brain, suggesting that this could be one aspect of the underlying cellular pathology (182). Consistent with this model, studies in neurons from *Mecp2*-KO or -cKO mice show both deficits in excitatory synapse development (17, 29) and failed activity-dependent maturation of GABAergic neurons (16). Neural activity is directly coupled to regulatory events on MeCP2 via calcium-induced protein phosphorylation at several sites including Thr308 and Ser421 (34, 181). Thr308 phosphorylation regulates MeCP2’s association with the nuclear receptor corepressor (NCoR) transcriptional repressor; thus, modulation of this site could change MeCP2’s ability to repress genes (87). The function of Ser421 phosphorylation remains unknown, but it is tightly tied to the elevation of intracellular calcium signaling (181), and mice bearing a nonphosphorylatable Ser421Ala mutation show impaired regulation of gene expression and plasticity induced by psychostimulants and antidepressants (30, 31, 39, 57, 66). Dysregulation of activity-induced gene expression has been reported in *Mecp2* mutant neurons; however, whether this is a direct effect of MeCP2 on these genes or a secondary consequence of synapse dysfunction is not clear (21, 112, 146). New studies continue to explore the transcriptionrelevant functions of MeCP2 in neuronal chromatin, including at enhancers (100), and these data may help to uncover roles for MeCP2 in transcriptional plasticity.

#### 3.1.3. RAI1 and the dysregulation of homeostatic plasticity.

Smith–Magenis syndrome (SMS) is a developmental disorder that is associated with altered learning and speech and language skills as well as distinctive facial features and sleep disturbances. SMS is caused by deletions in chromosome 17p11.2, where the causative gene is thought to be the TF retinoic acid–induced 1 (*RAI1*) (139). Duplication of this same chromosomal region causes a distinct phenotype called Potocki–Lupski syndrome that also includes features of ID and sleep disturbances (103). The dosage-dependent effects of *Rai1* mutations on learning and memory have been replicated in mouse models, supporting a role for RAI1 in both syndromes (9, 14). To identify plasticity functions and transcriptional targets of RAI1, Garay et al. (40) knocked down *Rai1* in mouse cortical cultures. Electrically silencing cortical cultures with the sodium channel blocker TTX drives a form of homeostatic plasticity called synaptic upscaling in which excitatory synapse strength increases to keep firing rates constant in the face of reduced excitatory drive (155). TTX treatment also induces a transcriptional program of inactivity-induced genes that is required for synaptic upscaling, though the mechanisms of this transcriptional regulation process remain poorly understood (20, 58, 86, 99, 132). Knockdown of *Rai1* mimics synapse upscaling and leads to a gene expression profile with significant similarity to that seen after TTX (40). Chromatin profiling studies show that RAI1 is bound across the genome at the promoters of activity-regulated genes and that it dissociates from chromatin following increases or decreases in neuronal activity. These data raise the possibility that RAI1 acts as a repressor to buffer the expression of a set of genes under baseline conditions such that those genes can become induced when TTX silencing causes RAI1 to unbind. This model offers new understanding of how changes in gene expression can couple to homeostatic plasticity and synaptic changes induced by decreases in neuronal firing.

#### 3.1.4. The transcriptional role of variants in the noncoding genome.

I have focused on rare variant mutations within coding sequence that have strong effects on ID because these mutations are easy to model with molecular genetics. However, the vast majority of genetic variants that contribute to cognitive function are outside of coding regions (96), where their consequences are more challenging to study. One way that noncoding genetic variation can impact gene expression is when variants fall within TF-binding sites in promoters or enhancers. Several studies have reported significant enrichments for variants associated with neuropsychiatric diseases within the set of activity-regulated enhancers identified in iPSC-derived neurons in culture (12, 127), though it is difficult to predict the impact of these variants on transcription without experimental validation. One study used natural variation between distantly related mouse strains to demonstrate that variants within and around enhancer-binding sites for Fos/Jun and their collaborating TFs can alter TF binding and the strength of enhancer activity (175). Moving forward, massively parallel reporter assays that test the function of enhancer variants in neurons (97) may help to identify transcriptionally important sources of noncoding variation that contribute to differences in the potential for cognitive plasticity.

## 4. CONCLUSIONS

The explosion of sequencing data over the past 20 years has substantially advanced our understanding of the molecular underpinnings of brain plasticity. From cataloging cell types to identifying activity-regulated gene-expression programs to discovering gene mutations that disrupt cognition in NDDs, sequencing data have facilitated the curation of a large set of high-confidence molecular mechanisms with functional contributions to plasticity. However, just as any single set of Legos can be assembled into more than one final structure, so too can different molecules work in distinct ways to support learning and memory. The stories told here show how molecular genetics strategies can test hypotheses of the relationship between specific gene products and changes in circuit function. The most significant takeaway is that the consequences of gene regulation for plasticity depend significantly on the cell type that has been activated as well as the circuit context of that cell. These data show how a sophisticated knowledge of molecular gene regulation can advance circuit-based understanding of brain mechanisms for learning and memory.

Despite progress, there is still much to learn. Although studies targeting excitatory versus inhibitory neurons reveal distinct functions of IEGs, these remain coarse categories of neuronal cell types, and future studies will need to narrow the focus on different subclasses of cells that have distinct functions in cortical circuits. Current transgenics largely rely on single genes to target specific cells, whereas cell subtypes are more commonly defined by the levels of expression of gene programs (114). Since cell type–specific programs of gene expression are determined by the accessibility of regulatory enhancers (52), using those same enhancers to drive transgene expression may help to focus the targeted expression of transgenes. Machine learning may also come into play to discover synthetic enhancers that can outperform endogenous sequences (64).

I have discussed the evidence that gene mutations associated with ID can impair plasticity via their impact on experience-dependent gene regulation. Given that increases in activity-dependent gene expression are correlated with memory, therapeutic strategies that enhance the expression of these genes might have the potential to improve cognition in neurological disorders. For example, histone deacetylases (HDACs) are enzymes that oppose the epigenetic activation functions of the transcriptional coactivator CBP. Interventions that inhibit or knockdown the HDACs drive increased histone acetylation at promoters and enhancers and have been associated with both elevated activity-dependent gene expression and improved memory in mouse models (41). Another option would be to directly elevate the expression of IEGs. IEG mRNAs are robustly induced following neuronal activity, but the proteins are normally rapidly degraded, restricting the time course of their action. Proteasomal degradation of IEGs and other nuclear TFs has been shown to selectively require an adaptor protein called midnolin (46), and KO of midnolin increases the amplitude and prolongs the time course of IEG expression. The challenge is to determine whether this would have a meaningful impact on adaptive plasticity, even if these approaches could globally increase activity-dependent gene expression, given the evidence that inducible gene expression programs have pleiotropic cell type–specific functions. Future studies that advance mechanistic understanding of activity-dependent gene regulation have the potential to offer additional approaches for cognitive enhancement.

## THE PROMISE AND PERILS OF USING IMMEDIATE-EARLY GENES TO DEFINE ENGRAMS

The induction of IEGs such as *Fos* is often used to conduct circuit mapping of experience-activated neurons in the central nervous system (CNS). Because reactivation of IEG-tagged ensembles can be sufficient to produce learned behaviors, these ensembles are often interpreted as memory engrams of the experience. Although *Fos* has been the most widely used activity marker, other activity-induced genes, including *Arc*, *Npas4*, and *Egr1*, have also been deployed as surrogate neuronal activity markers. Although all of these IEGs can function to regulate circuit plasticity, they do so via distinct molecular mechanisms. Furthermore, because the induction of each IEG is a response to the activation of distinct signaling cascades, the different IEGs are often found in physically distinct subsets of activated cells. One model proposes that this molecular heterogeneity of ensembles defines subengrams that store different features of the memory trace (178). Alternatively, the apparent differential expression of IEGs may only reflect whether the induction threshold for that gene was reached, which may or may not impact cellular plasticity. Studies that reactivate ensembles tagged by different IEGs in the same mouse will help to explore this concept (149).
